# Immune Responses to Dengue and Zika Viruses—Guidance for T Cell Vaccine Development

**DOI:** 10.3390/ijerph15020385

**Published:** 2018-02-23

**Authors:** Claude Roth, Félix G. Delgado, Etienne Simon-Lorière, Anavaj Sakuntabhai

**Affiliations:** 1 Functional Genetics of Infectious Diseases Unit, Institut Pasteur, 75015 Paris, France; fdelgadot@unbosque.edu.co (F.G.D.); etienne.simon-loriere@pasteur.fr (E.S.-L.); anavaj.sakuntabhai@pasteur.fr (A.S.); 2 CNRS UMR 2000–Génomique Évolutive, Modélisation et Santé, Institut Pasteur, 75015 Paris, France; 3 Virology Group, Universidad El Bosque, Bogotá D.C. 110121, Colombia

**Keywords:** Zika virus, dengue virus, T cell epitopes, vaccination

## Abstract

Despite numerous efforts to identify the molecular and cellular effectors of the adaptive immunity that induce a long-lasting immunity against dengue or Zika virus infection, the specific mechanisms underlying such protective immunity remain largely unknown. One of the major challenges lies in the high level of dengue virus (DENV) seroprevalence in areas where Zika virus (ZIKV) is circulating. In the context of such a pre-existing DENV immunity that can exacerbate ZIKV infection and disease, and given the lack of appropriate treatment for ZIKV infection, there is an urgent need to develop an efficient vaccine against DENV and ZIKV. Notably, whereas several ZIKV vaccine candidates are currently in clinical trials, all these vaccine candidates have been designed to induce neutralizing antibodies as the primary mechanism of immune protection. Given the difficulty to elicit simultaneously high levels of neutralizing antibodies against the different DENV serotypes, and the potential impact of pre-existing subneutralizing antibodies induced upon DENV infection or vaccination on ZIKV infection and disease, additional or alternative strategies to enhance vaccine efficacy, through T cell immunity, are now being considered. In this review, we summarize recent discoveries about cross-reactive B and T cell responses against DENV and ZIKV and propose guidelines for the development of safe and efficient T cell vaccines targeting both viruses.

## 1. History

Zika virus (ZIKV) is a Flavivirus transmitted by *Aedes* species mosquitoes. It is a single positive-stranded RNA virus closely related to yellow fever virus (YFV), dengue virus (DENV) and West Nile virus (WNV) [1]. First isolated in Uganda in 1947 [2], it remained confined to several regions in Africa and Asia from that time until the early 2000s. In 2007, however, it caused an explosive outbreak for the first time outside of Africa and Asia, on Yap Island, Federated States of Micronesia [3,4], followed by subsequent outbreaks with higher numbers of cases in 2013–2014 in French Polynesia and other South Pacific Islands and more recently in the Americas [5,6,7,8,9]. Although initially believed to only cause mild disease, the 2013–2014 and 2015 outbreaks in French Polynesia and Brazil clearly revealed that ZIKV causes neurological complications, such as Guillain-Barré syndrome in adults and microcephaly in infants born to ZIKV-infected women [10,11,12,13]. Phylogenetic studies indicated the presence of two lineages of ZIKV, the African and Asian lineages, the latter being responsible for the recent major outbreaks in French Polynesia and South America [14,15]. Notably, it was suggested that the enhanced infectivity of the Asian lineage of ZIKV was due to a spontaneous mutation in the gene coding for Non-Structural Protein 1 (NS1) leading to its higher secretion in the serum and infectivity to mosquitoes [16], which could explain its recent re-emergence in the Americas [14,15] despite its relative absence in South East Asia. More strikingly, several amino acid substitutions in the proteome or more specifically in the precursor membrane (prM) protein with possible functional implications for ZIKV biology and pathogenesis have been identified from ZIKV outbreak strains in South America [17,18]. 

In addition to the high infectivity of the Asian lineage in the Americas, one of the most important concerns today is related to the high level of DENV seroprevalence in areas where ZIKV is circulating [19]. This is particularly important given the structural similarities between ZIKV and DENV [20,21,22], and the existence of cross-reactive immune responses associated with disease pathogenesis [23,24,25,26,27]. Nevertheless, while it is now well established that a secondary infection with a heterologous DENV serotype represents a risk factor for the development of severe dengue disease, because of serotype cross-reactive or sub-neutralizing antibodies which can mediate antibody dependent enhancement (ADE) [28], it remains to be determined whether a previous DENV infection can also increase the risk of developing a more severe ZIKV disease in humans, as suggested by studies in mice [26]. Likewise, while ZIKV-immune plasma can enhance DENV infection in immune-deficient mice [24], the role of ZIKV immunity in protection or enhancement of dengue disease in humans is still unknown.

In this review, we address the most recent findings regarding the adaptive immune response against ZIKV, focusing on the effect of DENV pre-existing immunity on ZIKV infection, the underlying idea being to identify immunological parameters predictive of increased susceptibility or protection against ZIKV infection and disease. In this respect, we will review the current state of knowledge on the impact of anti-DENV antibodies on ZIKV infection and disease, and then summarize the recent data on the potential role of T cells in DENV and ZIKV immunity, with the aim to promote a long lasting immune protection against these two viruses.

## 2. Antibody Cross-Reactivity between Zika and Dengue Viruses

The high level of cross-reactivity among flaviviruses, especially DENV and ZIKV which share 54–59% sequence identity in the E protein [20,29,30], and their co-circulation in the same endemic regions have complicated serological approaches to discriminate between these two viral infections. In most cases, reverse transcription-polymerase chain reaction (RT-PCR)-based assays within a week post-infection, in combination with serological binding assays to recombinant proteins and functional neutralization assays in vitro, either by Plaque Reduction Neutralization Test (PRNT) or Flow-Cytometry-Based Neutralization Assay have been developed to distinguish between ZIKV and DENV infections [23,24,31,32,33,34].

Upon primary DENV infection, different types of antibodies have been found in polyclonal human sera: serotype-specific antibodies with strong neutralizing activity against the homologous DENV serotype, and cross-reactive antibodies with weak neutralizing activity and strong enhancing potential against heterologous DENV serotypes [35,36]. In secondary DENV infections, both type-specific and cross-reactive neutralizing antibodies, as well as cross-reactive enhancing antibodies to the other DENV serotypes, are elicited [37]. Following multiple DENV infections, the polyclonal response has been shown to contain mainly cross-reactive antibodies that recognize the different DENV serotypes, these antibodies being potentially associated with a more durable cross-protective immunity [37,38].

Upon ZIKV infection, it has been shown that the quality and type of antibody response depends on previous infection with DENV, with a more restricted specificity against ZIKV in DENV naïve individuals [24,25]. Using a panel of monoclonal antibodies (mAbs) derived from ZIKV-infected individuals with or without previous DENV immunity, it was shown notably that the antibody response against ZIKV includes envelope domain III (EDIII)-specific antibodies with strong neutralizing activity against ZIKV, as well as envelope domains I and II (EDI/II)-specific antibodies with high degree of cross-reactivity against the E protein of all four DENV serotypes and low neutralizing potential against ZIKV [24]. Thus, just like how EDI/II-specific and cross-reactive antibodies induced after DENV infection can enhance DENV and ZIKV infection [23,39,40,41,42,43], EDI/II cross-reactive antibodies raised by ZIKV infection can also potently enhance DENV and ZIKV infection [24,27]. Interestingly, not all cross-reactive antibodies are enhancing, as shown in a recent study where memory B cell clones derived from different ZIKV-infected individuals could produce antibodies that neutralize both ZIKV and DENV serotype 1 (DENV1) [44]. In this case, cross-reactivity was shown to result from a previous DENV1 infection and the clonal expansion of memory B cells specific of an EDIII epitope of ZIKV and DENV1 and neutralize both types of viruses. This observation provides the first evidence for a ZIKV-neutralizing antibody response that derives from pre-existing immunity to DENV. However, the low frequency of these cross-reactive memory B cells within the polyclonal population of circulating memory B cells raises the question of the impact of such responses in the immune protection against ZIKV infection. In this sense, it would be helpful to determine whether multiple DENV infections could boost such memory B cells with a broad spectrum of neutralization, in the same manner as the DENV1-induced cross-neutralizing response [44].

At the polyclonal level, it was confirmed that while a primary ZIKV infection could induce predominantly ZIKV-specific antibodies that poorly cross-react with the four DENV serotypes, a ZIKV infection in individuals with previous DENV infections results in the production of both ZIKV-specific and DENV-cross-reactive antibodies [45]. Interestingly, depletion of these DENV-cross-reactive antibodies did not affect the level of anti-ZIKV neutralizing antibodies, suggesting that ZIKV-specific neutralizing antibodies are produced after ZIKV infection regardless of previous DENV immunity [45].

Thus, while it is clear that DENV infections can induce the production of cross-reactive antibodies that recognize the four DENV serotypes, and to a lesser extent ZIKV, in most cases, they do not induce durable, high-level ZIKV cross-neutralizing antibodies [45].

Similarly, upon primary or secondary DENV infections, it is now well established that the level of DENV-specific neutralizing antibodies decay rapidly in the absence of re-exposure, or more slowly in endemic settings [46,47,48,49], and individuals with high neutralizing antibody titers have lower probability of symptomatic infections, in comparison with individuals with low neutralizing antibody titers [49]. In this sense, a positive correlation was clearly established between the low level of pre-existing anti-DENV antibodies and the severity of secondary dengue disease in humans, with a higher risk of severe dengue with anti-DENV antibody titers of 1:21 to 1:80 and a stronger protection against symptomatic dengue disease observed with higher antibody levels [19,50]. More recently and finally, while type-specific neutralizing responses were initially thought to elicit life-long immunity against homologous reinfections [51,52], there is now clear evidence that homologous reinfection can occur in the presence of serotype-specific neutralizing antibodies, leading to symptomatic disease [53,54]. While in some cases these homotypic reinfections have been observed more than 10 years after the initial infection [53], in other cases, such reinfections were identified with a shorter time interval of 1 to 2 years between the successive infections [54]. In both situations, the specific level of pre-existing anti-DENV antibodies provides an explanation for the homologous serotype reinfection and symptomatic disease observed in individuals from different endemic regions [53,54]. Although it is too early to determine whether the same correlation applies to ZIKV infection, it seems likely that the decay in the level of ZIKV-specific neutralizing antibodies in the absence of re-exposure to this virus should affect the outcome of future ZIKV infection and disease. 

## 3. T Cell Responses against DENV and Prospects for a Vaccine

Although the exact role of T cells during dengue virus infection and disease is still a matter of debate [55], there is currently a growing body of evidence supporting a protective role for T cells in dengue virus infection, both in human and mouse studies [56,57].

Briefly, similarly to the ADE phenomenon hypothesis associated to cross-reactive antibodies, it was proposed that DENV-specific T cells could play a detrimental role during secondary dengue infection. In this “original antigenic sin” scenario, an expansion of cross-reactive T cells with higher avidity to the previous infecting serotype would mask the specific T cell response against secondary infection, and would result in less efficient elimination of DENV-infected cells [58,59,60]. These cross-reactive T cells, stimulated upon a secondary infection with a different serotype, also displayed quantitative and qualitative differences in their response to the cross-reactive epitope [61], with higher ratios of Tumor Necrosis Factor alpha (TNF-α) to Interferon gamma (IFN-γ)-producing CD4 T cells [62], suboptimal degranulation but high cytokine production [63], or more specifically impaired IFN-γ production [64].

However, in spite of these studies, the direct demonstration of a pathogenic role of DENV-specific T cells in patients experiencing natural secondary dengue infection is still missing, and recent reports do not support a causative role for cross-reactive T cells in the pathogenesis of dengue hemorrhagic fever during secondary infections [65,66]. Indeed, increased frequencies of DENV-specific CD4^+^ and CD8^+^ T cells were detected in school children who subsequently experienced subclinical infection, in comparison with symptomatic secondary DENV infections [67]. In addition, protection or susceptibility to severe dengue disease has been associated with the expression of certain Human Leukocyte Antigen (HLA) molecules [68,69,70,71,72,73] and a beneficial function of CD8^+^ T cells against DENV infection was demonstrated after depletion of the CD8 T-cell compartment in interferon-α receptor knock-out mice (*ifnar^−/−^*) [74]. More strikingly, a strong correlation was established between protection against severe dengue and a polyfunctional memory CD8^+^ T cell response with a high magnitude in healthy dengue-immune individuals [75]. Finally and more recently, we observed a higher activation of Natural Killer (NK) cells and T cells in asymptomatic dengue viral infection; different T cell populations proliferate more and have an activated phenotype, with increased pathogen recognition, signal transduction and higher cytotoxic activity in asymptomatic DENV infected individuals compared to individuals with symptoms during DENV infection [76].

While CD4^+^ T cell responses are mainly directed toward the structural proteins capsid (C) and envelope (E) and the non-structural protein NS1, similarly to DENV-specific B cells that target prM, E and NS1, most CD8 T cell epitopes reside in the non-structural proteins NS3, NS4B and NS5, and to a lesser extent in the structural protein E [60,75,77,78,79,80].

Besides the identification of DENV-specific T cell epitopes recognized by activated T cells, these studies contributed to identifying correlates of protection, as the result of an efficient T cell activation by immunodominant peptides restricted by certain HLA alleles, and a robust and polyfunctional CD4 or CD8 response [75,81,82,83,84,85]. This is true, for example, for the HLA-B*0702 and -B*3501 class I molecules, and the HLA-A*0101 and -A*2402 associated with a high and a low CD8 T-response frequency and magnitude, respectively [75]. The same goes for the HLA-DRB1*0401 or -DRB1*0802 class II alleles, which are associated with an increased resistance or susceptibility to severe dengue disease, respectively, and the phenotype of responding cytotoxic CD4 T cells [81].

## 4. Identification ZIKV-Specific and DENV/ZIKV Cross-Reactive T Cells

In contrast to a large number of studies on T cell responses to DENV infection, to date, relatively little is known regarding the analysis of T cell responses to ZIKV infection. 

First predictions of ZIKV T cell antigens were conducted by modelling potential epitopes that could bind to different HLA class I or class II alleles, from the ZIKV proteome [86,87,88], and by identifying short peptides targeted by DENV-specific CD8^+^ T cells with conserved sequences between DENV and ZIKV [89], with the underlying assumption that these epitopes should stimulate cross-reactive T cells after sequential DENV and ZIKV infection. 

While such sequence conservation between several immunogenic CD8^+^ T cell peptides from DENV and the corresponding ZIKV sequence was highlighted in the E, NS1, NS3 and NS5 proteins [89], a study revealed that memory T cells against NS1or E proteins were poorly cross-reactive, even in donors previously infected by DENV [24].

**Figure 1:**
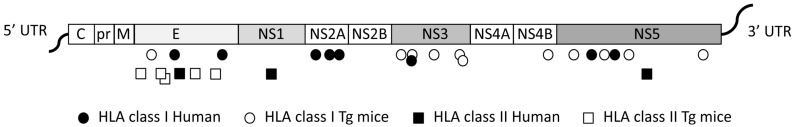
Schematic representation of class I- and class II-restricted cross-reactive Zika virus (ZIKV) epitopes identified in humans and transgenic mice. The ZIKV genome is not to scale. E: Envelope; HLA: Human Leukocyte Antigen; M: Protein M; NS: Non-Structural Proteins; pr: Precursor; Tg: Transgenic; UTR: Untranslated Region.

To identify first the dominant epitopes of ZIKV recognized by CD8^+^ T cells in the context of human HLA class I molecules, and to clarify the protective role of CD8^+^ T cells in ZIKV infection, different mouse models have been used. Type I interferon receptor-deficient mice expressing human HLA class I molecules (HLA-B*0702 or HLA-A*0101), which are susceptible to DENV and ZIKV infection, enabled identification of ZIKV peptides that are targeted by CD8^+^ T cells, and to show that dengue or Zika virus infections can induce the development of cross-reactive CD8^+^ T cells that are protective against ZIKV infection [90]. In HLA class II transgenic mice, CD4 immunodominant epitopes have also been mapped from ZIKV Envelope, and from non-structural proteins NS1, NS3 and NS5, among which several peptides in the envelope revealed cross-reactivity with other flaviviruses [91] (Figure 1).

In another mouse model, which lacks the type I interferon receptor in a subset of myeloid cells [92], adaptive transfer of ZIKV-immune CD8^+^ T cells was shown to inhibit primary ZIKV infection and replication [93]. Likewise, in *ifnar^−/−^* and in wild-type mice, adoptive transfer and depletion experiments have demonstrated that DENV-immune CD8^+^ T cells, but not DENV-immune sera can mediate cross-protective responses against ZIKV infection [94]. Although a correlation was previously established in mice and rhesus monkeys between the level of neutralizing antibodies induced upon ZIKV vaccination and the immune protection against ZIKV challenge [95,96], these studies highlight the essential role of CD8^+^ T cells in providing a protective immunity, in the presence or not of pre-existing DENV immunity, a finding that could help define future strategies of vaccine against DENV and ZIKV [97]. Finally, wild-type immunocompetent mice, *ifnar^−/−^* mice and Interferon Alpha Receptor (IFNAR)-depleted *rag1^−/−^* mice, have helped to characterize the phenotype of responding T cells after ZIKV infection and to demonstrate the protective effect of CD8^+^ T cells against ZIKV infection in various organs, including the brain and testes [98,99,100]. However, while these studies clearly demonstrate a major role for CD8^+^ T cells in controlling ZIKV infection, they all relied on experiments performed in mice with an altered immune system, a model that supports DENV or ZIKV infection but does not reflect the human situation. In addition, these mouse models do not take into account the human innate immune response against these flaviviruses, more specifically the NK cells that kill infected cells through the recognition of viral peptides in the context of HLA-C*0102 [101]. In summary, even though HLA transgenic mice expressing a single HLA allele do not mimic the human situation, the identification of immunodominant epitopes in these transgenic mice constitutes an important step towards preclinical evaluation of T-cell based vaccines.

For this purpose, to formally identify ZIKV-derived peptides in humans, and to determine whether a previous DENV infection can activate cross-reactive T cells, functional studies have been performed using human Peripheral Blood Mononuclear Cells (PBMC) collected from ZIKV endemic areas. From blood donors from different countries in Central and South America, the majority of the CD8^+^ T cell responses in DENV seronegative individuals were shown to be directed against structural proteins, whereas in DENV seropositive donors, a large proportion of CD8^+^ T cell responses were directed against the non-structural proteins with cross-reactivity for peptides in NS2A, NS3, and NS5 [102] (Figure 1). Strikingly, cross-reactive CD4^+^ and CD8^+^ T cells recognized peptides with identical or highly conserved sequences between DENV and ZIKV, with a higher magnitude of response, showing that DENV-specific memory CD8^+^ T cells can enhance the T cell responses to ZIKV. 

In light of the low protection observed in individuals DENV-seronegative at the time of vaccination with Dengvaxia, which lacks DENV non-structural proteins and fails to induce a competent T cell response [103], and taking into account the role of CD8^+^ T cells in preventing ADE [104,105] it is now becoming obvious that efficient vaccine candidates against DENV and ZIKV should be formulated to include CD4 and CD8 T cell epitopes, either alone, or in combination with B cell epitopes. 

Based on the recent identification of these T cell epitopes derived from ZIKV, work is currently ongoing to define a minimal antigen, which includes the most immunodominant peptides recognized by cross-reactive T cells, and which can induce a long lasting immune protection against DENV and ZIKV infection and disease.

## 5. Conclusions

Our understanding of the immune response to ZIKV has dramatically increased in the last years through in vitro studies with polyclonal and monoclonal antibodies from ZIKV-infected individuals, and through the identification of the epitopes inducing a strong memory and cross-reactive T cell response. While several animal models that could mimic the human situation have been developed to clarify the beneficial or detrimental role of the different immune mediators in disease protection, further phenotypic analyses of ZIKV-specific T cells, in asymptomatic or symptomatic donors will help define correlates of protection in natural immunity and vaccination against ZIKV infection and disease.
